# Cell freezing and the biology of inexorability: on cryoprotectants and chemical time

**DOI:** 10.1057/s41292-024-00331-4

**Published:** 2024-07-07

**Authors:** Hannah Landecker

**Affiliations:** Department of Sociology, Institute for Society and Genetics, University of California Los Angeles, 264 Haines Hall, Box 91551, Los Angeles, CA 90095-1551 USA

**Keywords:** Cryoprotectant, Cryoscience, Dimethyl sulfoxide (DMSO), Temporality, Toxicity

## Abstract

What can’t freezing hold still? This article surveys the history of substances used to protect cells and organisms from freezing damage, known as cryoprotectants. Dimethyl sulfoxide (DMSO) has since 1959 been the most widely used of these agents in cryopreservation. Here, its evolution from pulp and paper waste byproduct to wonder drug to all-but-invisible routine element of freezing protocols is used to trace the direct arc from protection to toxicity in theories of how and why cryoprotectants work, from the 1960s to today. The power of these agents to simultaneously protect and degrade is shown to reside in manipulation of chemical time via hydrogen bonding and electron exchange, thereby reframing freezing as a highly active and transformational process. Countering long-held assumptions about cryopreservation as an operation of stasis after which the thawed entity is the same as it was before, this article details recent demonstrations of effects of cryoprotectant exposure that are nonlethal but nonetheless profoundly impactful within scientific and therapeutic practices that depend on freezing infrastructures. Understanding the operationalization of chemical time in the case of cryoprotectants is broadly relevant to other modern technologies dedicated to shifting how material things exist and persist in human historical time.

Cryoprotectants have played a central role in historical and contemporary endeavors to freeze cells, seeds, tissues, and biological samples and substances for later use. It might seem that the two main protagonists involved in freezing are the freezer and the entity to be frozen. However, a third participant is inevitably needed: cryoprotectants, the topic of much concerted science and technological development. The assumption underpinning freezing in research, agriculture, conservation, or medicine is that a frozen entity should be stopped in time, and then start up again where it left off upon being thawed. Frozen sperm should go on to fertilize eggs, frozen DNA samples should maintain their sequence integrity, cells frozen in culture should start functioning and dividing again, eggs or embryos should be able to enter back into the process of reproduction without being affected by days, months or years spent suspended in a frozen state. These outcomes would not be possible without cryoprotectant agents, constituents defined not by their chemical structure but by what they do. They are substances which, “when added to cells in their medium, allows higher post-thaw recoveries than if it were not present” (Elliott et al. 2017, p. 75).

The role of cryoprotectants, despite being essential, is an overlooked and underappreciated element in the historical and social analysis of cryopreservation. Their investigation and elaboration exceed the question of a missing detail. The story helps us rethink frozen or vitrified life as a highly active state despite its dominant characterization as “paused or latent,” what Sophia Roosth calls “life not-itself” in her detailed genealogy of scientific observers fascinated by the appearance of “organisms that, in periods of suspension, do not do anything—they do not grow, metabolize, move, perceive, or respond to their surrounding environment” (2014, p. 59). In her account and others, technoscientific work on so-called latent or suspended biology is depicted as captivated by this paradoxical stasis, a pause in the incessancy of life that is not death—“bringing life forms to their limit, a point where all metabolic activity appeared to cease, only to warm them up again and return them to normal functioning” (Radin and Kowal 2017, p. 4).

The allure of stasis is practical as well as conceptual. Confidence in the ability to freeze and thaw at will has had profound effects across animal and human reproduction, the time and space of research, and the possibility of conservation and preservation of endangered species and historically significant biological samples. The idea of generating a physically latent state through freezing is conjoined to a second sense of latency, that of frozen biologicals as banked potential to “yield new knowledge” at a future date, unaffected by their time in storage (Radin 2013, p. 488). “Cryopower,” as Joanna Radin and Emma Kowal have characterized it, insists on its own exclusivity as the only way to “save” an embryo or a blood sample or a gamete of an endangered species for future life or knowledge (2017). Bringing the politics of the freezer to the fore, Radin shows that certainty as to the stasis of frozen things paradoxically limits the capacity of those who wield cryopower to imagine a different future: “Efforts to conjure future anteriority have trouble making space for the complexity of time and the unstable ontological status of objects through time” (2015, p. 363). What is in the freezer will not remain the same thing to the people who will engage with it at another point in time.

In this paper, I complement Radin’s important account of ontological inexorability by unearthing its equally underappreciated twin: biochemical inexorability. Paradoxically, biochemical inexorability arises from the very agents that enable cryobiology in the first place. That they are known a cryoprotective *agents* directs us to examine their activity, as the verb form of *protect* suggests. The frame of apparent standstill in freezing begins to fill with action when viewed through the lens of the positive set of ongoing events constituent to cryoprotection. Therein, the formation of hydrogen bonds with water alters its structure and viscosity, preventing the formation of damaging ice crystals. Antioxidant processes continuously—even in the frozen state—operate over the duration to lend electrons to free radicals that might otherwise damage the membranes, DNA, and proteins important to warmed-up entities’ viability upon thawing. In other words, stasis must be continuously kept up. It is life as maintenance, all the way down.

At the very same time the potency of cryoprotectants also carries with it a range of potentially untoward biological consequences. The metabolic and biophysical effects of cryoprotectants simultaneously underpin both protection and toxicity. Moreover, the procedures supported by cryoscience—research in cell biology, stem cell transplantation, conservation—go forward only with the entities that survive freezing and thawing, not those that perish and decay. These protocols may therefore be inadvertently selecting for the embryos or cells best able to withstand cryopreservation, i.e., the population that emerges is not the population that was frozen.

Digging into the specifics of cryoprotectant theory and development is to highlight what has been so hard to see: that these active processes of constructing stasis are simultaneously protective, selective, and degradative. The impression that some pure version of nothing is happening during the period of suspension actively obfuscates an observer’s ability to think about how being frozen and thawed might be a state and time of profound change, a period of activity after which nothing can be the same, rather than a suspension of activity bridging two identical states. After all, whosoever pays attention to the substance coating the tip of the poisoned spindle or the properties of the remediating spell when there’s Sleeping Beauty on the table taking up all the oxygen in the room, so to speak? Apparently completely unchanged between the moment she pricks her finger to the moment she opens her eyes, footnotes about the wisdom of doing follow up studies on her grandchildren don’t really figure in either that tale or in cryoscience. Nonetheless, the mere fact that the frozen entity is alive or moving after thawing or can be used to ten years later to inseminate a cow on the other side of the country and produce viable offspring doesn’t make it identical to the life from that went into the freezer some time before. To put it differently, just because you can do odd things *to* biological time doesn’t mean you get to escape being subject to the altered biological time you have generated.

An important outcome of reframing stasis as a process of maintenance is the foregrounding of a tradeoff between successful freeze–thaw processes and toxicity, a bargain that has rather silently been made with cryoprotectant usage. That is, “conflicting effects of protection versus toxicity” arise from the fact that the very same metabolic and biophysical effects essential to cryoprotectant mode of action are also inescapably consequential for the biology being “protected” (Elliott et al. 2017, p. 74). Of course, it might not matter much that we could be subtly shifting the epigenetic patterns of our offspring and of the reproductive materials of endangered species through widespread application of cryopreservation techniques. Perhaps changes to stem cells and seeds ensuant to exposure to cryoprotectants and cold are minor compared to the cost of losing them altogether. Perhaps the side effects of cryopreservative exposure during therapeutic transplants are small change in the tradeoffs of treatment for otherwise devastating diseases. Yet even if such changes do not become frankly pathogenic or damaging, shouldn’t we know about and think about them in some detail anyway as a different kind of history, rather than an escape from history? This would enable our capacity to consider freezing as an intentional compromise and a choice rather than an infallible solution enacting power over time (Edelstein et al. 2020). If what we are saving is an altered form of an endangered species, or if the technicity of human reproduction is changing the molecular being of humans—even in subtle ways—then it is important to delineate and theorize this generation of an overtly anthropogenic biology: a form of life whose form and function arise within the evolutionary and biochemical materiality of human history, not a form of life freed from history’s bounds (Landecker 2024).

The history of cryoprotectants is dominated by the biochemical materiality of one substance in particular, dimethyl sulfoxide (DMSO). As much as commentators have noted cold as infrastructure in everything from food distribution systems to assisted reproduction, DMSO is in turn infrastructural to cryopreservation across a wide range of freezing activities (Friedrich and Höhne 2016). This humble waste by-product of pulp and paper processing is a strikingly notable substance, often referred to in the scientific community as “the universal solvent,” because one can dissolve almost anything in it, including things that are not soluble in water (water being the other chemical compound that chemists frequently refer to as a universal solvent). Reference libraries of chemicals or pharmaceutical drug candidates come dissolved in DMSO; because it easily crosses membranes and other biological boundaries, it is often used as the “vehicle” through which a drug or toxicant is applied to an experimental animal subject. For example, if a researcher wants to test the effect of a hormone on behavior, they will dissolve the hormone in DMSO and then rub the mixture on the animal’s skin, because it will immediately cross the skin and be systemically distributed.

DMSO’s cryoprotectant capacities, as we shall see, are manifold, to the point that there are often no acceptable alternatives available for freezing protocols. Interviews with researchers in toxicology, chemistry, embryology, and conservation science about cryoprotectants quickly revealed the centrality of DMSO to a very wide range of practices in all these laboratories, and a mingled sense of danger and inevitability regarding the stuff. In terms of laboratory safety, the ability of DMSO to rapidly cross the skin carrying potentially toxic chemicals dissolved in it directly into the body makes practitioners very cautious around it. Yet these unique properties are also what makes it so useful and abundant. While this account keeps mostly to the documentary published record due to its commitment to detailing the history involved, my interlocutors pointed me decisively toward the frame of the story: the belated realization of a profound tradeoff in the use of powerful chemical substances for the maintenance of frozen life as an infrastructural element of contemporary science and reproductive medicine.

Like a torn nitrile glove, this article has admitted DMSO entry and now it has crept throughout, threatening to take over the narrative altogether. Following the lead of science studies scholar Joseph Dumit’s marvelous essay “Substance as Method” (2021), I am going to cede to this annexation, and first use DMSO as a vehicle to deliver to the reader a brief history of cryoprotectants. Understanding the evolution of *cryoprotectant theory* in this history makes the chemical activity involved in protection more legible as a challenge to assumptions of freezing as stasis. Second, highlighting the mode of action of DMSO allows me to examine the protection-toxicity bargain entailed in the use of cryoprotectant agents, and why their potential downsides were so hard to see for so long.

This study both complements previous work on the harnessing and mobilization of cold, and bridges between it and scholarship far beyond the “cryo” per se. The dark note of inexorability staining the optimism of cryopower could be seen as a singular story of cell freezing, but it is better situated as one among other moments of reckoning with the “anthropochemicals” that have become intrinsic to contemporary existence (Papadopoulos 2022). This account of cryoprotectants connects the social scientific study of cryoscience to the call for better elaboration of “Anthropocene elements”: small material stories of chemical scale and temporality that compose bigger stories of capitalism and toxicity (Neale et al. 2022). In turn, within a rapidly evolving scene of critical study of the residues of industrial chemistry (Boudia et al. 2021), the tale of cryoprotectants brings the manipulation of chemical time and the historicity of the bargain between protection and toxicity inherent in its control to the foreground for analysis.

## Cryoprotectant Discovery and Theory

The terms *cryoprotectant* and *cryoprotective agent* (CPA) were settled upon by consensus at the second Annual Meeting of the Society for Cryobiology in 1965 with the recognition that the survival of freezing by animal cells “almost always requires specific treatment of the cells with at least one chemical agent” (Karow 1969, p. 209). Indeed, it is not clear that there would have even been a society for cryobiology in 1965 and surging interest in the uses and underlying science of freezing in biology were it not for the widely lauded though rather accidental discovery in the late 1940s that glycerol added to the medium in which avian sperm was being frozen meant a much higher survival and viability rate post-thawing than any other techniques available at the time (Polge 2007; Polge et al. 1949). The findings about glycerol were analyzed by James Lovelock, then a chemist and Polge’s colleague at the National Institute for Medical Research at Mill Hill, London. Lovelock hypothesized that cells were injured chemically during freezing, by the osmotic loss of water from inside the cell to ice forming outside of it, resulting in an increased electrolyte concentration in the cell. He showed that the protective effect of glycerol depended on its entry into the cell, surmising that other “neutral solutes of low molecular weight which are non-toxic and which can permeate the cell should also protect it” (Lovelock 1954, p. 266).^1^

Lovelock worked with biologist Marcus Bishop to test this prediction with a newly commercially available candidate, dimethyl sulfoxide (DMSO). They used cattle red blood cells, which were not permeable to glycerol. The keen interest and financial involvement of American commercial cattle breeders in the push to preserve bull semen by freezing was no doubt also behind the choice of species for the experiment, indicated by historical context rather than the paper itself (Radin 2015). Where a two hour equilibration with glycerol gave only slight protection against hemolysis (bursting open) in the freeze–thaw process, it only took 30 s of equilibration with DMSO for “complete protection” (Lovelock and Bishop 1959).

The discovery of the protectant properties of glycerol and DMSO were “watershed” moments for cryobiology (Radin 2017, p. 36). Much of the research that ensued was empirical, in the sense that the “chemical individuality” (Garrod 1902) of every cell type from every species entailed a slightly different protocol in terms of being appropriate to lipid profiles, internal compartmentalized distribution of cellular water, and intrinsic antioxidant capacity—blood cells were quite different from spermatozoa, cattle cells were different from chicken cells. There was also much tinkering with the speed of freezing and the volume of cell suspensions to be frozen. It was work pursued on the foundation of an assumption that the technique worked, and what lay ahead was the task of optimizing it.

Optimization was the common aim across very varied domains of cryoprotectant development. For example, in freezing food everything used had to be safe for human consumption, and taste and appearance rather than viability was at stake. Nonetheless, it became absolutely routine to understand the choice of carbohydrate and/or antioxidant cryoprotectant as the key determining factor in optimizing the look and edibility of frozen and thawed meat, fish, and vegetables (MacDonald and Lanier 1991). More recently the shelf-life of lyophilized (freeze-dried) probiotics has encountered the same need to incorporate antioxidants and cryopreservatives to enact active “stabilization” against the “slow but inescapable decrease” in bacterial activity (Romero-Bachiller and Santoro 2022, p. 820). Very high concentrations of cryoprotectants have been essential to the increased use of cell vitrification in assisted reproduction and plant preservation, a very rapid cooling that solidifies biological material into a glass state without crystallization (Rall and Fahy 1985). Yet even here the attention has been mostly on the drama of the speed of transition from one state to the other and the claims for its enhanced efficacy over previous freezing methods thanks to the prevention of damaging ice crystals (Benson 2008; Lafuente-Funes 2023).

As things do when they become part of protocols, cryoprotectants quickly lost their novelty, becoming unremarkable elements of projects whose drama seemed to come from elsewhere, as has been richly noted in the burgeoning literature on the social, medical, and economic impacts of cell freezing for human reproduction, clinical care, conservation, and research material storage evidenced by the papers in this volume. Empirical tinkering with cryoprotectant concentrations, mixtures, and freezing rates ensued. Nonetheless, despite this rapid routinization, there was simultaneously a minor but continuous line of activity from Lovelock’s work to the present articulating *cryoprotectant theory*, a concerted effort to understand the principles of their effects rather than the details of their application. In this body of work there are three main characteristics of cryoprotectants in general, and DMSO in particular, that stand out as explanatory frameworks for understanding the protective function of these substances: (1) how they interact with and change the structure of water, (2) their antioxidant properties, and (3) a capacity to permeate. This theoretical work is dominated by DMSO simply because it “is the cryoprotectant of choice for most animal cell systems since the early history of cryopreservation” (Awan et al. 2020).^2^

First, interaction with water structure is perhaps the most intuitive of these principles of cryoprotectant action, as the watery basis of life is well known. Statements about the body being 70% water tend to depict it inaccurately as “a structureless, space-filling, background medium in which biochemical events occur,” as water is an equal partner in constant interaction with everything in it (Watterson 1988, p. 101). The body’s molecules are dissolved in water and are supported by it, or in more technical parlance, “the changing, subtle, multifaceted configurations that water molecules can assume under physiological conditions are necessary to solvate and stabilize the full gamut of essential biomolecules” (Elliott et al. 2017, p. 75). Water molecules comprise one oxygen atom and two hydrogen atoms, and many specific characteristics of fluid water and solid ice are determined by how these molecules stick to one another or stick to other substances because of hydrogen bonding and polarity. At different concentrations, cryoprotectants enhance or disrupt the structure of water, and thereby its interaction with everything in the cell. Glycerol and other alcohols such as ethanol and methanol are polar but less so than water, while DMSO is strongly polar. If you dissolve DMSO in water, any given water molecule in the solution will preferentially interact with (make hydrogen bonds with) the oxygen in a DMSO molecule rather than another water molecule.

One effect of the rearrangement of the structure of water by cryoprotectants is to depress the freezing point of the solution; at higher concentrations they can prevent the formation of ice entirely in the transition from liquid to solid state at very low temperature, the principle behind vitrification. While all this talk of structure suggests a very static set of alternative molecular configurations, chemical theories of water have leaned since the 1970s toward the idea that “water exists as transient but interrelated networks of ‘flickering clusters’ momentarily constrained by hydrogen bonding and continually reorganizing on a picosecond timescale” (Elliott et al. 2017, p. 75). Cryoprotectants enter, and shift, the flickering intermolecular temporality of the interaction of water with itself and between water and membranes and proteins. Thus, while retardation or slowing down in a freezing protocol is often framed as the *loss* of activity or motion on the part of the frozen entity, at an atomic and molecular level these changes in state and rate of molecular interactivity are more accurately depicted as an alteration, a change in rate of reorganization of both internal and external water driven by the intervention of the cryoprotectant.

The second principle of cryoprotectant action is a capacity to constantly buffer against the damaging molecular effects of cellular stress. Again, it is worth emphasizing that the most dominant molecule in living organisms is water, and it is not inert—and neither are the cryoprotective agents chosen specifically because of their chemical interruption of the structure of water. The outsize number of water molecules relative to any other molecules constituting a body and its metabolism means that chemical reactions are not sitting around going equally back and forth in equilibrium, but are dominated in the direction of hydrolysis, the breaking down of a compound in reaction with water (Danchin and Sekowska 2015). It is little wonder then that enormous physiological and biochemical stress occurs in cells being frozen, as water is actively being withdrawn into ice from all these cellular processes.

For example, changed concentrations of salts and other solutes causes osmotic stress, as dissolved solutes are excluded from forming ice and the cell contents literally become more concentrated. There is the stress of the constituent enzymatic processes slowing and not providing the usual substrates or failing to function as usual in controlling metabolic waste products. In a situation in which some cells are dying or undergoing acute stress, their fragmented membranes or their cellular distress signals will spill out into the medium and expose other cells to more free radicals or cue onward cellular stress responses. The protective action of the cryoprotectant in this case has to do with remediating the damaging effects of free radicals such as hydroxyl (*OH), molecular fragments that have an unbalanced number of electrons and are therefore highly reactive. Thus DMSO is known as an *OH “scavenger.” Antioxidant substances can lend an electron to free radicals without themselves becoming destabilized and stop what can become chain reactions of damage that compromise the lipid structure of membranes or the integrity of DNA structure.

Third, the capacity to permeate points to the continuous interaction of the cell with its environment, and the compartmentalizing and organizing effects of cellular membranes. Only agents that can slip through or be actively transported into the cell can enter the relations detailed above. Cryoprotectants operate within these temporal and spatial domains of atoms and electrons, in which biological and chemical time come together and are meaningful in each other’s terms as metabolism. As with the relationship to water molecules, antioxidant action is dynamic, manifold, ongoing, and continuous, occurring at the scale of electrons and the time of oxidation–reduction reactions (the term for any reaction in which the partners in the reaction change in their electron count and therefore their charge). It is the active maintenance of the unbroken state of important molecules and structures, from DNA to membranes.

## The (strange) story of DMSO

Noted in the brief account of Bishop and Lovelock’s 1959 demonstration that DMSO could be used where glycerol could not, the rather astonishing capacity of DMSO to penetrate membranes quickly and completely without obvious damage to them—to cross biological barriers without breaking them—was an early standout property of this substance. While not all cryoprotectants enter cells, DMSO does so with speed and thoroughness, and trials at different temperatures and concentrations only accentuated the sense that what took minutes or hours or never occurred with glycerol took seconds with DMSO, making it much more “practical” to use (Bickis et al. 1967).

As it enters the cell, we can see what the scientists at that time saw: an agent that inhabited cells with astonishing rapidity to protect them on the inside. In addition, we might add another overlay to this image, and see a rather literal displacement of what is naturally there—a biochemical relation of water and life evolved over millennia—by a substance possessed of a distinctly twentieth century historicity. Freezing, a procedure so often depicted as a suspension of time, might then be productively recast as a total permeation of the cell with the historicity of industrial society, a physical shoving aside of natural-historical time by human historical time as DMSO displaces water.

Historians of chemistry have made clear how modern chemical ideas and theories were developed in materials that came to the bench for very specific reasons and at very particular times because they were important to mining, industry, to food production, or to pharmaceutical work (Klein and Lefèvre 2007). This same sense of material historicity can be brought to bear on Lovelock and Bishop’s small research report, occupying a mere page in *Nature* in 1959 reporting on what seemed at the time to be a minor useful extension of the fortuitous discovery of glycerol as a cryopreservative agent. The reason that DMSO was newly on the supply shelf, available to be tested in this way, was that it was derived from lignin, a component of wood removed in paper production.

The Crown Zellerbach company, a pulp and paper conglomerate based in San Francisco, opened a Chemical Products Division in 1955, and shortly thereafter dedicated a facility in Bogalusa, Louisiana to recovering other commodity chemicals from the “kraft black liquor” separated from cellulose fibers in the making of paper (Goheen 2018). This byproduct is voluminous—about seven units of black liquor are produced for every unit of cellulose fiber pulp—and the aim of the new factory was to recover and sell organic chemicals and energy from these wastes. Dimethyl sulfoxide was foremost among these, but a market had to be generated for it. Crown Zellerbach therefore did in-house chemistry to develop uses of the substance and marketed it aggressively, with “applicability as a solvent, in the manufacture of synthetic films and fibers, in the manufacture of paint and varnish removers, in the formulation of resin, wax and lacquer products, as a medium for gas recovery and separation, as a softener and humectant for cellulosic materials, as a reagent, reaction medium and diluent and for a host of other uses,” as the company’s patent on the process of its production put it (Goheen et al. 1960). By 1967, DMSO was being referred to as “one of the most intensively researched chemicals to engage the interest of science this century,” in part because it was a promoter of faster chemical reactions; it “allows the inherent reactivity of the dissolved material to show through, speeding up many millions of times” (Seidel 1967, p. 34).

As sociologist Phillip Davis noted in 1984 in his comprehensive study of DMSO in terms of social movement theory, the use of DMSO in cryobiology sparked a whole other career for the substance as a controversial “wonder drug” (Davis 1984).^3^ Stanley Jacobs, a medical researcher in surgery at the University of Oregon Medical Center who was interested in organ freezing and had read the 1959 Lovelock and Bishop paper, approached Crown Zellerbach about working with DMSO and thereby met a company staff chemist, Robert Herschler, who had been tasked with investigating commercial uses for the new product, using it to carry dissolved pesticides into trees.

The finicky details of freezing protocols fell quickly by the wayside as Jacobs and Herschler pursued what seemed to them the extraordinary capacity of DMSO to quickly penetrate the skin without damaging it. Application to the outside of the body was followed within a matter of minutes by a strong taste in the mouth and a distinctive garlic-like aroma on the breath, indicating very fast penetration of the skin and systemic spread into the tissues. Having applied it in large amounts to laboratory animals and finding it to be of apparently low toxicity, they then proceeded to apply it to all kinds of people and situations in what from a contemporary perspective looks like a horrifyingly cavalier manner: a laboratory assistant’s sprained ankle, a University of Oregon football player with a headache. Jacobs hypothesized that DMSO could carry drug therapies across the blood–brain barrier to “treat” people with Down’s Syndrome.

Alarming both the medical research community and the drug regulatory community in the United State, Jacobs and Herschler happily shared their beliefs with the media, talking about DMSO as a cheap and non-toxic panacea for treating everything from sprains to intellectual disability, and the anecdotes of miraculous treatment quickly spread, ending up on the front page of the *New York Times* in 1963 and *Time Magazine* in 1964 under enthusiastic headlines such as “DMSO: New Wonder Drug” (Kerr 1964). Jacobs and Herschler published two reports on DMSO as a “new concept in pharmacotherapy” in *Current Therapeutic Research* in 1964, and Crown Zellerbach received a Claimed Investigational Exemption for a New Drug on the strength of the animal safety data they produced, allowing limited human testing. The company then licensed six large pharmaceutical companies to study the safety and efficacy of DMSO for treating inflammation, relieving pain, inhibiting bacterial growth, relaxing muscles, carrying other drugs into the body or increasing other drug efficacy, and the potential uses of its apparently extraordinary ability to cross membranes including the blood–brain barrier.

DMSO was accordingly tested on prisoners at Holmesburg Prison by University of Pennsylvania dermatologist Arthur Kligman, in one case a 24-week study in which DMSO was applied daily on the bodies of 20 inmates from chin to pelvis, studies that brought first the attention and then the sanction of the FDA, which had only in 1962 altered its procedures for human testing in light of news of thalidomide causing birth defects (Hornblum 2013, p. 56; Visperas 2022). A frustrated editorial in the *Journal of the American Medical Association* pronounced DMSO “as abundant as the Pacific Ocean” and decried its trial by media first and science a distant second (JAMA Editors 1965, p. 120). Jacobs was charged with bribing an FDA official in a case that ended with a hung jury and a mistrial in 1982. In the end, the FDA withdrew approval for human testing due to animal data that showed retinal damage from DMSO exposure, and DMSO was dropped by the drug companies. It is currently only used therapeutically as a vehicle for topical treatments and in the treatment of bladder pain in interstitial cystitis, generating a robust set of conspiracy theories about suppression of this wonder drug. The substance continued (and continues) to be sold without restriction as a solvent and has a significant community of users who employ it to self-treat a wide range of conditions.

None of this controversy had much effect on the use of DMSO as a cryoprotectant because freezing protocols did not involve direct application of DMSO as a drug to human beings. There was no equivalent to retinal damage at the level of the cell. Toxicology was centered on adverse outcomes in animal models or mutagenicity assays that would pick up carcinogens that caused direct damage to DNA (Creager 2014). Indeed the strange conjunctures that arise with the DMSO stories may seem like side notes—admittedly fascinating ones, but perhaps not relevant to the topic of “cryogenic life in contemporary societies,” under discussion in this special issue (Lemke 2021). What should we make of these events in which the future author of the Gaia hypothesis (Lovelock), working in a research milieu on questions framed by the interests of cattle breeders in freezing sperm, introduces a newly commercially available solvent to cryobiology that promptly becomes embroiled in a human subject testing scandal and conspiracy theories about the FDA keeping inexpensive drugs from the population despite their cure-all properties?

These stories may seem only randomly associated. Lovelock’s next publication in *Nature* concerning DMSO came thirteen years later, when it was discussed as a gaseous product of algal metabolism emitted from the oceans, a planetary sulfur cycle that was part of his conception of how living organisms generate the conditions for biotic life in a homeostatic feedback loop (Lovelock et al. 1972). Lovelock and Lynn Margulis then published the statement of the Gaia hypothesis in 1974, in which they laid out the idea that the Earth’s atmosphere was not just “a mere environment for life,” but a “component part of the biosphere,” and that all living things taken together as an ensemble dynamically regulate the chemical composition and climate of their own existence—sulfur is metabolized and chemically rearranged by organisms as much as it forms the abiotic environment in which they live (1974, p. 2).^4^ In ensuing discussions and debates, Lovelock’s earlier experience with cryoscience is rarely even mentioned.

Nonetheless, it seems to me that there is more to say about the turn to Gaia theory and its relation to the turn taken in the story of cryoprotectants that I am about to recount. While Lovelock never engaged directly with the particular protection-toxicity bargain that is now becoming clear in the context of cryobiology with DMSO, the stories bear structural similarity and not just biographical coincidence. Lovelock turned away from using chemical principles of sulfur compounds in pursuit of cryoprotectant efficacy in the name of furthering productivity aims in the logging and cattle industries, toward chemical principles as the foundation for understanding the “revenge of Gaia”—how the self-regulating homeostasis of planetary sulfur cycles are perturbed by industry and agriculture (Lovelock 2007). Gaia theory and cryoprotectant theory alike are directly transformed in and by the material wake of the massive industrial production of biologically consequential chemicals. Both respond to the ‘alterlife’ made visible and urgent in terms of systemic planetary or cellular impact exactly because of the mobilization at scale of these chemicals—the scale of their production becomes with time the scale of their impact (Murphy 2017).

Yet there was nothing so dramatic as acid rain or climate change to make the biotic impacts of DMSO in the cell legible, nor is there a charismatic theorist to point to in the history of cryobiology calling for change. Only a slow realization of compromise accompanied by an uneasy sense that the very means that enable cryopreservation in its current form are also the source of its problems.

## Spelling out the protection-toxicity bargain

In this section I trace out a brief history of conceptualization of cryoprotectant toxicity, through its long conscription to the task of optimizing outcomes, to a paradoxical result of the success of that venture: the more they are used, the more evident their long-term effects beyond protection become. Let us work up to the present stepwise, returning first to 1959. Lovelock and then a raft of investigators following on his finding were focused on finding a substance that would work, and whose efficacy could be optimized. It took a full thirty years for any significant turn from a theory of why cryoprotectants work to a theory of why they don’t work, i.e., cryoprotectant toxicity, despite the FDA’s withdrawal of approval for human experimentation with DMSO. While these protocols continued (and continue) to fall below one hundred percent recovery rates of frozen cells, the focus was not on the ones that burst open or stopped moving or didn’t go on to yield live offspring, much less more subtle changes to cell function or long-term heritable effects visible in populations. It was on success rates; the ability to resuscitate a good percentage of frozen cells was taken as the sign of the technique’s power, in which failures would be overcome with inevitable improvements.

To understand this obdurate focus on power rather than fallibility, and the excited confidence of the press that a wonder drug with no caveats was a likelihood, it is useful to remember that substances such as DMSO—participant in the widespread onward valuation of industrial waste—was a potent carrier of what Kim Fortun calls the “language ideology” that structured a twentieth century political economy of chemical commerce (2014, p. 213; Liboiron 2021; Romero 2021). Within this ideology, she writes, “the focus is on what works”:

It is an essentialist, functionalist logic that privileges what goes on inside bodies, products, and fence lines, orienting research, business, and law. It assumes that things are what they are intended to be—that they are their essence—and nothing more: Chemical plants produce chemical products for use (and sale), without polluting emissions. Pesticides kill insects, but pose no harm to other bodies and ecologies. Production is protected; pollution is externalized. The perspective is overwhelmingly positive (2014, p. 313).

This analysis is a precise one for the description of cryoprotectants in the research and technical literature between the 1950s and the 1980s, and to a certain extent still today. The next step in the story occurs in the 1980s, when a few biologists voiced early concern about this overweening focus on success, arguing that cryoprotectants were in and of themselves demonstrably toxic, and the mechanisms of that toxicity had gone largely unstudied. Their observations give voice to just how hard it was to see the price of these techniques in those decades: “The protective effects of cryoprotective agents are so useful and impressive that comparatively little attention has been focused on the negative effects of these chemical agents as they are used by cryobiologists.” There were thousands of reports in the literature on the “biochemical effects of cryoprotectants” yet “very few that help us to understand cryoprotectant toxicity” (Fahy et al. 1990, pp. 247–248).

It is not coincidental that Greg Fahy, one of the scientists calling to fellow cryobiologists to pay attention to cryoprotectant toxicity and its mechanistic basis in membrane and organelle damage, dehydration, and biochemical interaction with key cellular enzymes, is also credited with establishing the foundations for vitrification of organs and reproductive cells in the mid-1980s and commercializing that process (Rall and Fahy 1985). Vitrification employs much higher percentages of cryoprotectant to avoid ice formation than the prior slow freezing protocols. Rather ironically, its successful deployment depended on extensive work by Fahy and others to understand the molecular basis of cryoprotectant toxicity so they could then attempt to neutralize it by adding yet more agents. “The ability to reduce the toxicity of a penetrating cryoprotectant by adding more penetrating cryoprotectant is a rather amazing one, and it has been very important for the development of minimum-toxicity solutions” (Fahy 2010). To our understanding of *protection* as active chemical work occurring in the interaction of cryoprotectant, water, and cellular components, these more complex freezing media concoctions reveal another layer of action: cryoprotectant agents acting upon one another and disabling various organismal self-protection mechanisms.

By the end of the 1990s, a more elaborated understanding of cellular stress was emerging in the broader context of the life sciences, alongside a molecular explanation of stress-triggered cellular signals initiating programmed cell death (Baust et al. 2000). This explained why “success” rates in terms of cellular viability could look good in the hours directly after thawing, but then decline significantly over the next couple of days: the stress of thawing can initiate what is known as apoptosis, or “biomolecular-based cell death” that manifests “many hours post-thaw” (Baust et al. 2009, p. 91). Such understanding of the interaction between freezing and basic cell biology led to the addition of molecular inhibitors of the apoptotic cascade to the arsenal of cryoprotectants. However, as with the antioxidant “neutralizers” described above, none of these fixes is complete, and cell types, tissues, and organisms are quite variable in their capacity to survive these procedures even with the ever-increasingly complexity of the cocktails of agents applied to them.

What these more recent cryoprotectant recipes demonstrate is that even from their beginning, theories of cryoprotectant toxicity were enrolled in the larger project of optimization. This brings us to the apprehensive situation of the present day, in which a renewed round of questions is being raised about the biological effects of cryoprotectants beyond the stark binary parameters used to date in these optimization projects—viable/non-viable, living/dead, motile/non-motile or replicating/non-dividing. These parameters have equated survival or viability with sameness. As one commentator rather acerbically put it in a review of effects of cryopreservation on genome stability in frozen cells, “Unfortunately, concerns about cryopreservation have tended to focus simply on the survival and viability of cells following the cooling and thawing processes, the assumption being that having survived the process and resulted in a live birth, the cryopreserved sample or tissue was in essence completely identical to its ‘fresh’ state” (Kopeika et al. 2015, p. 210). These assumptions of before-and-after sameness, as I have noted above, rely on the idea that *nothing happens* in the cell or organism in the time of being frozen. Theories of cryoprotectant action, and their inseparability from cryoprotectant toxicity, by contrast raise the important question of what freezing can’t hold still: what proceeds biochemically, nonetheless. “Contrary to the belief that cryostorage can effectively ‘stop time’, there is some evidence to suggest that cells may still ‘gain’ age throughout the freezing–thawing cycle” (Kopeika et al. 2015, p. 210).

The very success of cryoprotectants in pursuing these previous parameters of optimization is driving a renewed set of hard questions about the preservation-toxicity bargain in which the preserving effects and the toxic effects are not necessarily different from one another. Indeed, it is the very power of the agent to preserve that also is the driver of a shifted biology compared to what existed before freezing. Again, while these questions bear on all cryoprotectants and on the physical effects of freezing itself such as changes in cell volume, they tend to be most acute when it comes to DMSO, because of its ubiquity, chemical singularity in terms of properties, and pivotal role. While discussions of replacing DMSO increase in proportion to the numbers of indications that it has many non-lethal but nonetheless consequential deranging effects on the cells, tissues, and embryos that are preserved in it, the research community has a frank understanding that there is currently no alternative.

In recent years, the concept that it will be possible to replace DMSO in cryopreservation media while still maintaining good post-thaw recoveries has been discussed [but] a wide application for such an approach has yet to be achieved. … It must be recognized that at the present time, the vast majority of applied cryopreservation practices depend upon the efficacy provided by DMSO for high functional capacities after thawing. (Awan et al. 2020, p. 1482)

Unlike the previous era of controversy about DMSO toxicity that seemed only relevant to direct application of the substance to patients, now egg and embryo freezing, expanding human marrow and organ transplantation networks, and aspirations for using stem cells in regenerative medicine concern hundreds of thousands of individuals and medical encounters (Cromer 2018; Liburkina 2022; Waldby 2015).

If the assumption that nothing happens during freezing is incorrect, then what *does* happen? This question has both a short simple answer and a long complicated one. Because they interact with and displace water, cryoprotectants can impact everything that is shaped by water in the cell: protein folding, DNA conformation, membrane permeability. Much more complicated is the nature of the evidence of these manifold impacts and their consequences for frozen reproductive cells, for the basic research produced from cell lines processed with standard freezing protocols, and for the patients treated with cells and tissues that have gone through freeze–thaw before implantation. Scientists looking at cells in culture, using sophisticated measures now available for visualizing everything from gene expression to changes in the epigenetic modification of DNA and chromatin, note what they call “drastic alterations” in treated cells compared to untreated controls (Verheijen et al. 2019).

Others testing early life DMSO exposure in an in vivo rodent model see profound changes to neurological physiology and function in the exposed animals, observing: “Despite short-term exposure at low, putatively nontoxic concentrations, DMSO led to changes in behavior and social preferences, chronic alterations in glial cells, and changes in essential regulatory brain metabolites” (Rabow et al. 2021, p. 1). Of what significance are new studies that state matter-of-factly that “DMSO is a non-inert substance with significant effect across macromolecules and subsequently cellular functions and molecular profiles,” including the ability to alter membrane lipid composition, DNA conformation, and cellular proteins’ function and stability? (Baldelli et al. 2021). Even to the non-scientific reader it should be evident that such language implies that the short answer to “what does DMSO change in cell biology and function,” is that it changes everything.

It is of course not the job of the critical social scientist to adjudicate whether the right metric for measuring the methylation landscape of the epigenome was used in one study, or to try and adjudicate whether animal model systems are relevant to human conditions. Rather it is to situate these questions within the longer history presented above. What is of significance is not that this study or that has demonstrated changed epigenetic profiles in cells after cryoprotectant exposure, but the *form* that questions are taking, and the way investigators are framing these studies. The first notable characteristic of this form is the highlighting of the heretofore unthought character of the profundity of biological effects of substances that have become so widely used they are all but invisible. As with gestalt switches, you either see it and it is legible to you, or you failed to notice it at all, for years: “it is difficult to understand how it could be biologically plausible that reproductive cells or embryos subjected to the completely unnatural process of cryopreservation would have absolutely no structural or functional response to it,” yet there are vanishingly few long-term studies to understand the full range of these effects (Kopeika et al. 2015, p. 222). Indeed, it takes a great deal of both rhetorical and evidentiary work to get investigators to rethink cryoprotectants as highly agential, after decades of their description as inert, non-toxic antifreezes that are cheap, widely available, and classified into low-risk regulatory categories.

A second notable characteristic of these studies is that they are beginning to occupy a space in between viability and death, in between capacity to fertilize and yield live birth and developmental failure. Researchers are measuring parameters that are not life and death, but altered signaling pathways, shifted protein configuration, the balance of oxidant and antioxidant molecules in the cellular economy, or membrane density. “With the passage of time,” notes one senior scientist recounting decades of work in the field, “we have come to understand that the functional competence of spermatozoa cannot be defined merely in terms of the ability of these cells to fertilize an oocyte; it also needs to incorporate an assessment of their ability to program a normal pattern of embryonic development” (Aitken et al. 2016, p. 1). What if, these researchers imply, the harder question at play in cryopreservation between success and failure is a life subtly but irrevocably altered? What if, instead of causing death, freezing causes genome instability and changed gene expression patterns that will then repeat across the ensuing cellular and generational lifetimes that flow from that embryo or gamete? What if freezing is not a suspension, but a swerve whose trajectory takes the specific form of late industrial life, permeated by the confidence that chemicals act only as they are intended to (Fortun 2012)?

A third notable characteristic of recent studies directly examining the biological impacts of cryoprotectants is the subset concerned not directly with patient safety or human developmental impacts, but with the onward trajectory of the knowledge enterprise itself. It is common, for example, to cryopreserve the cell cultures used in *in vitro* panel screening of drug candidates. The cells are standard components of these testing systems, often ordered up from a commercial vendor and “stored” frozen until needed. If one is screening for drug effects on a particular cellular pathway relevant to lung cancer, immortalized lung cancer cell lines or even cancer patient-derived primary cultures are often used, and freezing is part of the infrastructures by which cells move around between countries and laboratories, storage facilities and active use (Delvenne et al. 2023). Moreover, these water-insoluble drug candidates are often dissolved in DMSO, and the drug structure itself can be contorted by its solvent. DMSO could therefore be shaping what the investigator sees using this system, at each of its steps. Yet the “broad off-target effects of DMSO on protein signaling networks” with a focus on these drug target pathways is little studied; a lone report recounts that even ultra-low concentrations of DMSO caused widespread changes to proteins in the key networks targeted by cancer therapies in eight commercially available cell lines (Baldelli et al. 2021, p. 13).

In short, we may conclude that cryoprotectants are not agents of stasis when viewed through the lens of recent theorization of their biological impact. Their propensity for activity and the changes they enact during the freeze–thaw process have been all but invisible for decades, occluded by an ideology of optimization packaged in a set of success metrics focused on binary categories of life or death, motility or immobility, viability or sterility. Little concerted investigation has been trained on the very large spectrum of alteration that potentially exists between these poles. The emergence of epigenetic, post-translational, protein-focused technologies of measurement of alteration is beginning to provide an adjustment to the previous set of rougher measures of success, such as percentage of cell survival, or whether chromosomes show clear evidence of breakage or rearrangement. Greater detail and increased computational power have enabled more detailed comparisons between the before-and-after state in terms of upregulation or downregulation of gene expression or patterns of DNA and histone methylation that show a wide range of subtle changes arising from freezing and cryoprotectant use.

The exact implications of these detected alterations for human life and health, or for the robustness of knowledge systems dependent on freezing, remain unclear. Also murky is whether the behemoth of accepted practice, assumed harmlessness, and the cheapness and ubiquity of these reagents will be shifted in the least by this rather belated realization of the protection-toxicity bargain, when the outcomes remain in the realm of subtlety rather than mortality. It is in one sense too late; the infrastructure and the capacities are already built with these agents, particularly DMSO, and because they are constitutive of the very ability to work in this way, attempts to swap them out with something else have not gotten far. In another sense, it is the character of optimization that the solution always lies ahead and therefore it can never be too late; these alarmed registrations of epigenetic change and protein-signaling deformation may well simply end up enfolded in the quest for new protocols that are just a bit better than the old, and the hardest questions about the very basis of the endeavor of freezing eggs and embryos and marrow and seeds in light of the biology of inexorability are never confronted head on.

## Conclusion

The first part of this article dug into the short career of cryoprotectant theory, noting that the apparent power of molecular protection through the freeze–thaw cycle derives from interactions with water, antioxidant properties, and the capacity to permeate the cell. Mechanistic explanations of how these substances change the structure of cellular water and enter into the interactions between water and the biomolecules of the cell point toward a constant activity on the part of the cryoprotectant taking place at the level of hydrogen bonding and electron transfer, quelling ice formation and stopping the free radical chain reactions leading to damaging peroxidation in membrane lipids and other cellular structures. This analysis might leave us with the idea that the "work” cryoprotectants do is tiny-scale ceaseless maintenance of the status quo. However, the flip side of such potent characteristics—that they alter all these chemical relations without causing death—is that these protective capacities are also the basis of alteration. Toxicity is not a different event going on in another part of the cell from protection, involving different mechanisms. Rather, the upside and the downside come together, as is made clear by later tinkering with cryoprotectant recipes in which more cryoprotectants were added to protect cells against the negative effects of the initial cryoprotectants.

I have argued in the second part of this article that despite their depiction as a release from the inevitable march of time, acts of cryopreservation depend on flooding the cell with human social history. Into cellular metabolism, the long-evolved mechanisms of the compartmentalization and ordering of chemical reactions, comes silvicultural chemical waste, dreams of therapeutic panaceas devoid of drawbacks, and ideologies of optimization. Close analysis of how their protective capacities are also, simultaneously, their toxic capacities allows a more circumspect understanding of the full range of cryopreservation practices as fundamentally transformative ones rather than exercises in stasis.

At the same time this analysis may be useful in a broader set of considerations about cryoscience as one example among others of the social manipulation of chemical time. By highlighting and historicizing the nature of the protection-toxicity bargain struck with cryoprotectants, in particular dimethyl sulfoxide (DMSO)—perhaps the most widely used solvent across the life and biomedical sciences today—this article has sought to bring a complementary and rather unexplored perspective to the sociohistorical study of the science, technology and social impact of cryobiology, one that should inform the discussion of freezing, but also goes beyond it. Thinking about a substance such as DMSO and how it works brings one to a domain that encompasses cryobiology and preservation, but also resituates it in the broader frame of what we might think of as “chemical time,” an important but so far rather silent partner to the framework of biological time that has understandably loomed rather larger in social scientific studies of biotechnology (Poleykett and Jent 2023; Stevens 2024).

Just as chemistry deals at the spatial microscale of atoms and electrons, there is a very specific micro-temporality in operation in the living chemistry of anything biological, frozen or not. From cryopreservation to continuous-process manufacturing in industry, the sequence and timing of chemical reactions is both the target of technological intervention, and the locus of the effects of those interventions on human social and historical time. Whether altering the chemical structure of water or driving polymer formation forward, the time of bonding and free radical reactivity is the domain of manipulation and effect. Recently, DMSO was found to be capable of breaking down per- and polyfluoroalkyl substances (PFAS), or “forever chemicals” at reasonably low temperature and pressure (Trang et al. 2022). That the same substance could both enable and degrade forms of apparent obdurate timelessness is another way to indicate that what is at stake in querying the cryoprotectant is an understanding of cryobiology and states of freezing as the very active manipulation of chemical time in the construction of stasis, a technoscientific endeavor of profound economic and social consequence.

Understanding this operationalization of chemical time in the case of cryoprotectants may shed light on the many other modern technologies dedicated to shifting how material things exist and persist in human historical time: the many accelerants, antioxidants, and emulsifiers participating in the viscosity, permeability, and degradability of modern material culture, and the very broad spectrum of anthropogenic life generated in the ensuing unremarked space of ongoingness that persists somewhere between the polarities of technological success or failure.

## Data Availability

A data availability statement is not applicable for this article.

## Footnotes

1 The authoritative source on the early history of cryobiology and the many events that came together to constitute these technologies, including the use of other industrial by-products such as carbon dioxide from fermentation and lime-making, is Joanna Radin’s *Life on Ice* (2017).

2 DMSO has also been similarly crucial to plant cell systems because few other cryoprotectants are able to as effectively cross both the plant cell wall and the cell membrane.

3 The historical account I give here relies on the meticulous documentation and timeline provided by Davis’s article published in *Social Problems*. Davis not only collected all the relevant media coverage and regulatory hearing transcripts, but he also interviewed individuals making a living selling DMSO as a health remedy, and individuals who were using DMSO to self-treat pain or cancer. While this piece seems to have attracted very little attention (at the time of this writing it has been cited only 19 times since its publication in 1984), it really deserves re-reading in light of the various spurious drug treatments such as ivermectin that emerged in the course of the Covid-19 pandemic.  For a more extended consideration of DMSO as generating a form of ‘anthropogenic biology’ see (Landecker 2024).

4 The Gaia Hypothesis has become a topic of much discussion lately, as geobiologists, climate scientists, evolutionary biologists, and science and technology studies scholars revisit, debate and historicize the idea that the Earth is a living organism generating its own homeostatically regulated conditions, as Lovelock put it (Doolittle 2017; Latour and Lenton 2019; Lovelock 1988; Stengers 2017). This debate is outside of the scope of this article, except to note that Lovelock’s early contributions to cryobiology do not figure in how anyone interprets or analyses his later work.
